# Tumor growth and angiogenesis is impaired in CIB1 knockout mice

**DOI:** 10.1186/2040-2384-2-17

**Published:** 2010-08-30

**Authors:** Mohamed A Zayed, Weiping Yuan, Dan Chalothorn, James E Faber, Leslie V Parise

**Affiliations:** 1 Department of Pharmacology, University of North Carolina at Chapel Hill, Chapel Hill, NC, USA; 2 Department of Biochemistry & Biophysics, University of North Carolina at Chapel Hill, Chapel Hill, NC, USA; 3 Department of Cell and Molecular Physiology, University of North Carolina at Chapel Hill, Chapel Hill, NC, USA; 4 Current Address: Department of Surgery, Division of Vascular Surgery, Stanford University Hospital & Clinics, Stanford, CA, USA; 5 Current Address: Institute of Hematology, Chinese Academy of Medical Sciences, Tianjin, China

## Abstract

**Background:**

Pathological angiogenesis contributes to various ocular, malignant, and inflammatory disorders, emphasizing the need to understand this process more precisely on a molecular level. Previously we found that CIB1, a 22 kDa regulatory protein, plays a critical role in endothelial cell function, angiogenic growth factor-mediated cellular functions, PAK1 activation, MMP-2 expression, and *in vivo *ischemia-induced angiogenesis. Since pathological angiogenesis is highly dependent on many of these same processes, we hypothesized that CIB1 may also regulate tumor-induced angiogenesis.

**Methods:**

To test this hypothesis, we allografted either murine B16 melanoma or Lewis lung carcinoma cells into WT and CIB1-KO mice, and monitored tumor growth, morphology, histology, and intra-tumoral microvessel density.

**Results:**

Allografted melanoma tumors that developed in CIB1-KO mice were smaller in volume, had a distinct necrotic appearance, and had significantly less intra-tumoral microvessel density. Similarly, allografted Lewis lung carcinoma tumors in CIB1-KO mice were smaller in volume and mass, and appeared to have decreased perfusion. Intra-tumoral hemorrhage, necrosis, and perivascular fibrosis were also increased in tumors that developed in CIB1-KO mice.

**Conclusions:**

These findings suggest that, in addition to its other functions, CIB1 plays a critical role in facilitating tumor growth and tumor-induced angiogenesis.

## Background

Pathological angiogenesis is a hallmark of tumorigenesis and is a discrete event in solid tumor formation [1,2]. Unlike physiological forms of angiogenesis that occur during embryonic development and normal wound healing, pathological, tumor-induced angiogenesis often ensues due to imbalances in either angiogenic activators or inhibitors [1,3]. Recent pre-clinical and clinical studies have employed strategies to target tumor vasculature as a means to impede tumor growth and eventual metastasis [4,5]. However, thus far monotherapy with such agents in human subjects has been limited due to marginal effectiveness, toxicities, and relative lack of specificity [6,7]. Therefore, further exploration is currently underway for more specific drug targets that are not essential for physiological forms of angiogenesis.

CIB1 is a 22 kDa EF-hand-containing regulatory protein that was originally identified as a binding partner for the cytoplasmic tail of the platelet integrin αIIb, and later found to inhibit αIIbβ3 activation in megakaryocytes [8,9]. Subsequently however, CIB1 was found to be widely expressed in various organs, tissues, and cell types, which suggested that it likely has additional uncharacterized roles [10,11]. Accordingly, we and others have demonstrated that CIB1 binds to and regulates the activity of various proteins including the transcription factor PAX3 [12], the polo-like kinase Fnk and Snk [13], the inositol 1,4,5-triphosphate receptor [14], Rac3 [15], PAK1 [16], and FAK [17]. Among these binding partners, PAK1 and FAK are known to regulate endothelial cell (EC) function *in vitro*, and contribute to angiogenesis *in vivo *[18-20]. Therefore, this connection led us to investigate whether CIB1 can also play a role in EC function and various forms of angiogenesis.

Recently, we reported that CIB1 is not required for developmental angiogenesis, as CIB1-KO mice are viable and female mice are fertile [9]. However, CIB1 is essential for proper EC signaling and functions such as migration, proliferation, and nascent tubule formation [21]. Loss of CIB1 in ECs also leads to attenuated responses to angiogenic growth factors such as VEGF and FGF-2, which result in decreased expression of the zinc-requiring matrix-degrading proteinase MMP-2. Moreover, CIB1-KO mice are impaired in pathological as well as adaptive forms of ischemia-induced angiogenesis, and PAK1 and ERK1/2 activation are significantly decreased in CIB1-KO ECs and ischemic muscle tissue [21]. Although these data clearly demonstrate that CIB1 participates in ischemia-induced angiogenesis *in vivo*, its role in other forms of pathological angiogenesis, like tumor-induced angiogenesis is still unclear.

As in ischemia-induced angiogenesis, tumor-induced angiogenesis is highly dependent on angiogenic growth factors and MMPs [22-24]. Accordingly, tumor allografts in knockout mice for growth factors such as FGF-2 and MMPs such as MMP-2 and MMP-9 exhibit significantly reduced tumor growth and tumor-induced angiogenesis [1,25,26]. Since our previous studies demonstrated that CIB1-KO mice have decreased growth factor-mediated angiogenesis, and CIB1-KO ECs have attenuated MMP-2 expression, we hypothesized that CIB1-KO mice may also have a defect in tumor-induced angiogenesis. To test this conjecture we allografted CIB1-KO mice with murine tumor cells that endogenously expressed either very low levels (B16 melanoma tumor cells) or very high levels (Lewis lung carcinoma cells) of MMP-2, as previously demonstrated by zymography analysis [25]. We report here that, in addition to regulating tumor growth, CIB1 in the host significantly contributes to tumor-induced angiogenesis.

## Methods

### Allografts

Male WT and CIB1-KO mice between 15 and 18 weeks old were anesthetized with 1.125% isoflurane supplemented with 2:3 oxygen-air. Rectal temperature was closely maintained at 37.0 ± 0.5°C. Hair was removed from the hind-limb ventral adductor thigh region using depilating cream, with care to avoid erythema. Mice were given a single subcutaneous injection of either 5 × 10^5 ^B16 melanoma cells (purchased from UNC-CH Tissue Culture Facility) or 2.5 × 10^5 ^Lewis lung carcinoma cells (ATCC, Manassas, VA) that were impregnated in 50 μL of 50% growth factor reduced Matrigel. Tumor allografts were grossly inspected 14 days after tumor cell injections, and photographs were obtained to document differences in tumor morphology between WT and CIB1-KO mice (*n *= 8-11 mice per group). Gross tumor necrosis was defined by superficial discoloration of the tumor. Fourteen days after tumor cell injection, tumors were isolated from animals, and were weighed and then immersed in water to estimate the tumor volume.

### Tumor fixation, histology, and capillary density assessment

Tumors isolated from animals were fixed in 2% PFA for 48 h, with a solution change at 24 h. Tumors were rinsed in water, placed in 70% ethanol for 48 h with shaking and another change of solution at 24 h. Four biopsies (~2-3 mm in size) were obtained from both the center and periphery of each tumor and embedded in paraffin. At least 3 interrupted sections, 6 μm thick and 50 μm apart, were obtained and stained with either H&E, or Masson's trichrome (MT), a marker for fibrosis-associated collagen deposition[27]. For each tumor, one random interrupted section of all four biopsies was assessed for the incidence of necrosis or intra-tumoral bleeding, spanning 70% of the biopsy tissue section. Similarly, one random interrupted section of each biopsy was assessed for the incidence of perivascular fibrosis.

To assess capillary density, the plasma membranes of capillary ECs in tumor sections were labeled with Alexa Fluor 594-conjugated isolectin GSL-1-B4 (1:100; Invitrogen), or with primary rat anti-mouse CD31 (PECAM-1) antibody (1:50; Pharmingen) and subsequently Alexa Fluor 594-conjugated secondary goat anti-rat IgG (1:1000; Invitrogen). Using a Nikon TE2000U inverted fluorescent microscope with an OrcaER, four high magnification immunoflorescent and H&E images were collected for each biopsy to yield a total of 16 images per tumor specimen. Using ImageJ software, the number of vessels with a clear lumen and < 7 μm were counted by an independent observer, who was blinded as to animal genotypes. Tumor microvessel density was reported as the average number of microvessels per high-magnification frame.

### Statistical Analysis

Continuous variables were compared with the Student's *t *test. We considered *P *< 0.05 to be significant.

## Results and Discussion

Almost all tumor cells secret angiogenic growth factors and MMPs (such as MMP-2 and MMP-9) to recruit and stimulate new blood vessel formation [1,28]. Since we reported previously that CIB1-KO mouse tissue and ECs have reduced growth factor-induced angiogenesis and MMP-2 expression [21], we asked whether CIB1-KO mice also have a defect in tumor-induced angiogenesis. To address this question we subcutaneously injected the adductor hindlimb regions of WT and CIB1-KO mice with B16 melanoma tumor cells (which are known to secrete angiogenic growth factors such as VEGF, FGF-2, PDGF, but only very low levels of gelatinases MMP-2 and MMP-9), and Lewis lung carcinoma tumor cells (which, in addition to secreting various angiogenic growth factors, secrete higher levels of MMP-2 and MMP-9) [29].

**Figure 1:**
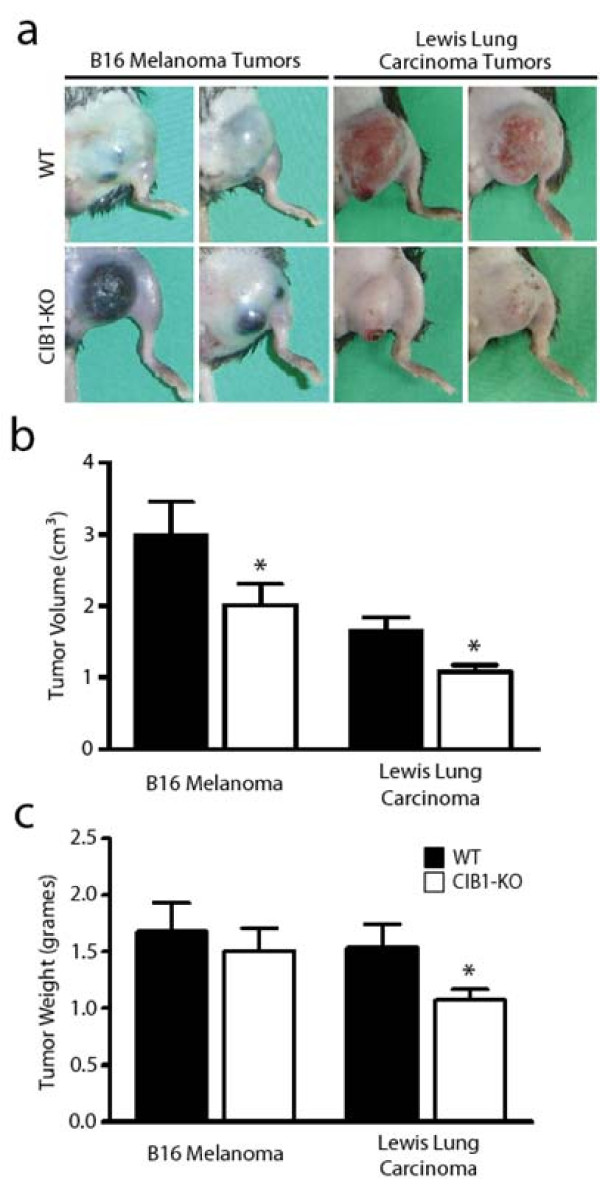
**Allograft tumors in CIB1-KO mice are smaller and have a distinct morphological appearance**. (a) Two representative images of B16 melanoma tumors or Lewis lung carcinoma tumors that developed after 14 days in either WT or CIB1-KO mice. Tumors in CIB1-KO mice had a distinct morphological appearance, with gross necrosis of melanoma tumors and blanching of carcinoma tumors. (b, c) Tumors that developed in CIB1-KO mice were smaller in volume and weight compared to WT mice, but no significant difference was noted for melanoma tumor weight. Error bars are ± SEM (n = 8-11 mice per group; * *p *< 0.05).

Fourteen days after tumor cell injection, signs of melanoma tumor necrosis were evident in a large subset of CIB1-KO mice (Figure 1a). Tumors in approximately 64% of CIB1-KO mice demonstrated evidence of gross morphological necrosis compared to 33% of WT mice (Table 1). This observation prompted us to immediately terminate the study and harvest allografted tumors from WT and CIB1-KO mice for further analysis. Gross dissection of melanoma tumors from CIB1-KO mice revealed that a large subset of these tumors were composed of highly necrotic fluid-laden tissue, and surrounded by small pockets of bleeding. In contrast, melanoma tumors extracted from WT mice were more homogenous and dense, and surrounded by less areas of bleeding. Furthermore, melanomas extracted from CIB1-KO mice were significantly reduced in volume (32% decrease, *p *= 0.05), but not weight (8% decrease, *p *= 0.34; Figure 1b and 1c). The decreased volume and increased gross necrosis of melanoma tumors in CIB1-KO mice suggest that these tumors were not as well sustained metabolically and therefore were not growing as rapidly as melanomas in WT mice. In part, this difference may be explained by the relative lack of MMP-2 in the tumor and peri-tumor micro-environment in CIB1-KO mice, leading to reduced tumor angiogenesis, thus mimicking the reported phenotype of reduced allograft tumor growth and angiogenesis in MMP-2-KO mice [25].

**Table 1:** Melanoma tumors have more gross necrosis in CIB1-KO mice.

Mouse Genotype	Total Number of Mice	Mice with Tumor Necrosis	Mice with No Tumor Necrosis	% of Mice with Tumor Necrosis
B16 Melanoma Tumors^a^				
WT	9	3	6	33.3%
CIB1-KO	11	7	4	63.6%^b^

Lewis Lung Carcinoma Tumors				
WT	8	0	8	0.0%
CIB1-KO	9	0	9	0.0%^c^

^a^Tumor necrosis was grossly evident in B16 melanoma tumors that developed in both WT and CIB1-KO mice.

^b^The number of tumors with clear evidence of gross necrosis in CIB1-KO mice was statistically significant (p = 0.05), chi-square test with one degree of freedom.

^c^There was no evidence of gross necrosis in Lewis lung carcinoma tumors that developed in both WT and CIB1-KO mice.

Interestingly, unlike the allografted melanoma tumors, none of the Lewis lung carcinoma tumors that developed in CIB1-KO and WT mice demonstrated evidence of gross morphological necrosis (Table 1 and Figure 1a). Compared to melanoma tumors, the complete lack of gross necrosis in carcinoma tumor in CIB1-KO mice may be due to differences in the physiology and cellular metabolic demand of Lewis lung carcinoma cells. Alternatively, this difference may be attributed to the ability of Lewis lung carcinoma cells to express and secrete high levels of MMP-2, which does not occur as effectively in B16 melanoma cells. Nevertheless, similar to melanoma tumors in CIB1-KO mice, Lewis lung carcinomas were also 30% reduced in weight and 35% reduced in volume when compared to these carcinomas in WT mice (*p *= 0.02 and 0.008, respectively; Figure 1b and 1c).

Moreover, carcinomas in CIB1-KO mice also appeared more blanched (Figure 1a), which suggested that these tumors were not as well perfused. To measure perfusion in Lewis Lung carcinoma tumors we used a non-invasive Doppler flowmeter for periodic assessment of tumor perfusion in real-time. Doppler perfusion of melanoma tumors in CIB1-KO mice was not assessed since they appeared to be grossly necrotic in appearance. Doppler images of 14 day old carcinomas demonstrated areas of clearly higher tumor perfusion in WT, but not CIB1-KO mice (areas in white; Additional file 1: Figure 1a). Quantification of these data also demonstrated a trend of reduced carcinoma tumor perfusion in CIB1-KO mice. However, due to variations in tumor dimensions and orientation, no overall statistically significant differences were extrapolated from this analysis (Additional file 1: Figure 1b). Nevertheless, like melanoma allografts, the decreased size and differences in carcinoma allograft gross appearance in CIB1-KO mice suggest that these tumors were not growing as efficiently as carcinomas in WT mice.

**Figure 2:**
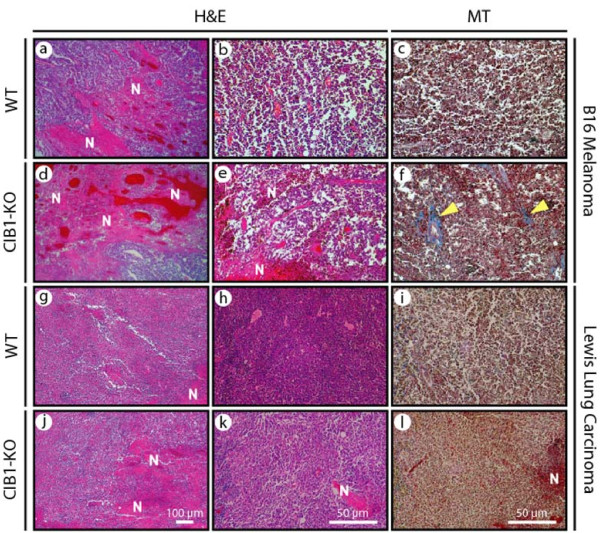
**Increased microscopic necrosis and bleeding in CIB1-KO allograft tumors**. Fourteen days after tumor cell injection, tumors were isolated from animals and fixed. Sections were sectioned and stained with H&E or Masson's trichrome (MT) as described in Methods. Representative images were captured at two different magnifications using a Nikon D100 camera attached to a Nikon inverted microscope. Representative low magnification images (a, d, g, and j), and high magnification (b, c, e, f, h, i, k, and l) of melanoma and carcinoma tumors. Areas of necrosis (N) and bleeding are noted on H&E-stained sections (a, b, d, e, g, h, j, and k). Fibrosis stained in blue (identified with yellow arrows) is noted on MT-stained sections (c, f, i, and l).

To further assess the allograft melanoma and carcinoma micro-environments we obtained biopsies from allograft tumor peripheries and centers. To minimize sample bias, biopsies were obtained from random locations and we histologically examined random areas of each biopsy section. Histological examination at low and high magnification of melanoma biopsies from WT and CIB1-KO mice revealed areas of tumor necrosis and intra-tumoral bleeding. However, melanoma biopsies from CIB1-KO mice contained larger areas of tumor necrosis and intra-tumoral bleeding (Figure 2a, b, d, and 2e). Similarly, intra-tumoral and perivascular fibrosis, associated with tissue injury and fibrosis, was more frequently found in melanoma tumors in CIB1-KO mice (Figure 2c and 2f). Although these differences in perivascular fibrosis, and intra-tumoral necrosis and bleeding (that span at least 70% of a biopsy's surface area) were not statistically significant in melanoma tumors, the incidences were higher in CIB1-KO mice compared to WT mice (*p *= 0.37, and 0.11 respectively; data not shown). Similar examination of carcinoma tumor biopsies from CIB1-KO and WT mice also revealed distinct areas of intra-tumoral necrosis and bleeding; however differences in the overall incidence of this between CIB1-KO and WT mice were less pronounced compared to those noted for melanoma tumors (Figure 2g, h, j, and 2k). Minimal perivascular fibrosis was observed in carcinoma tumors in both WT and CIB1-KO mice (Figure 2i and 2l). These histological observations are consistent with our gross observations, and therefore further suggest that in an MMP-2-deficient micro-environment in CIB1-KO mice, melanoma tumors are less viable and have significantly impaired growth.

**Figure 3:**
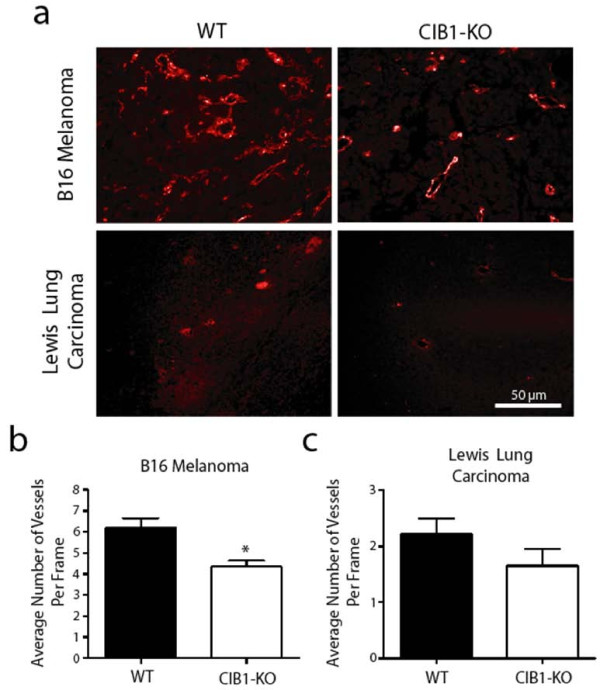
**Melanoma in CIB1-KO mice have reduced vascularization**. (a) Representative immunoflorescent images of Isolectin GSL1-B4-stained melanoma tumor sections and PECAM-1-stained carcinoma tumor sections. The number of vessels with a clear lumen and < 7 μm in melanoma (b) and carcinoma (c) tumors were counted by a blinded observer. Tumor microvessel density is reported as the average number of intra-tumoral microvessels per frame. Error bars are ± SEM (*n *= 7 - 11 mice per group; * *p *< 0.05).

Reduced tumor size and increased tumor necrosis observed in CIB1-KO allografts may also be due to reduced tumor-induced angiogenesis that is necessary for efficient delivery of oxygen and essential nutrients to the proliferating tumor cells. Thus, we assessed the microvessel density in sections obtained from biopsies of melanoma and carcinoma allografts that developed in both CIB1-KO and WT mice with either PECAM-1 or Isolectin GSL1-B4. Isolectin GSL1-B4-stained microvessels were noted in melanoma tumor sections, and similarly PECAM-1-stained microvessels were noted in carcinoma tumor sections (Figure 3a). The PECAM-1 antibody did not stain microvessels in melanoma tumors, and isolectin GSL1-B4 stained Lewis lung carcinoma cells (data not shown). At high magnification we observed that the microvessel density in melanoma tumors in CIB1-KO mice was 29% less than that observed in tumors isolated from WT mice (*p *= 0.002; Figure 3b). Correspondingly, the microvessel density in carcinoma tumors in CIB1-KO mice trended lower, although this potential difference did not reach a p value < 0.05 (26% decrease, *p *= 0.08; Figure 3c). Therefore, these data demonstrate that not only do allografted melanoma tumors in CIB1-KO mice have increased incidence of necrosis, but they also have reduced intra-tumoral neovascularization.

The decreased microvessel density in allografted tumors in CIB1-KO mice may be due to decreased expression and secretion of MMP-2 by CIB1-KO ECs, which we reported previously [21]. Itoh *et al*. (1998) demonstrated that MMP-2-KO mice have decreased tumor growth and metastasis. In accordance with our observations, the study also reported that MMP-2-KO mice have significantly decreased B16 melanoma and Lewis lung carcinoma allograft tumor growth. Since this decrease in tumor growth was largely unaffected by the difference in expression of MMP-2 by melanoma and carcinoma cells, the authors concluded that host-derived MMP-2 has a critical role in tumor progression and angiogenesis. Likewise, melanoma tumors in CIB1-KO mice have significantly decreased tumor-derived and host-derived MMP-2. Minimal MMP-2 would at least in part explain the decrease observed in allograft tumor growth and neovascularization in CIB1-KO mice. In addition, it would also suggest an alternative explanation for why a higher incidence of gross and histological necrosis was noted in melanomas, but not in carcinomas that have high levels of tumor-derived MMP-2 and low levels of host-derived MMP2.

The combination of increased tumor necrosis and decreased tumor-induced angiogenesis in CIB1-KO mice may also be a direct result of attenuated EC signaling in these mice. As previously reported, ECs deficient in CIB1 have reduced PAK1 activation. The activation of this serine/threonine kinase is an important point of convergence between growth factor- and integrin-induced signaling that is mediated by small GTP-bound GTPases such as Cdc42 [30,31]. Leisner et al. demonstrated that CIB1 binds to PAK1, thus preventing its interaction with Cdc42 and subsequent activation in various cell types [16]. Since PAK1 primarily mediates its effects on ECs via the Ras-Raf-ERK1/2 MAPK signaling pathway [18], we previously tested whether MAPK signaling is affected when CIB1 is not present. ERK1/2, but not p38, MAPK signaling was significantly attenuated in CIB1-KO ECs *in vitro*, and in CIB1-KO ischemic and non-ischemic muscles *in vivo *[21]. Thus, a CIB1-mediated inhibition of the PAK1-ERK1/2 pathway may explain the decrease noted in tumor-induced angiogenesis in CIB1-KO mice. Further studies are currently underway to determine if PAK1 downstream targets such as MMP-9 and TIMP-1 are also affected by levels of CIB1 expression.

There may also be alternative mechanisms by which CIB1 mediates its effects on tumor growth and neovascularization. We have observed decreased monolayer resistance in CIB1-KO ECs (unpublished data), suggesting that CIB1 is necessary for proper EC monolayer permeability *in vitro*. Related to this observation, in preliminary analysis of gene array studies we found that PECAM1 was among the most significantly downregulated genes in CIB1-KO ECs (*p *= 0.017, unpublished data). Not only is PECAM1 important for maintaining EC monolayer integrity, but it also plays an essential role in tumor growth and metastasis [32,33]. For example, local administration of anti-murine antibodies targeting EC-expressed PECAM1 inhibits both growth factor- and tumor-induced angiogenesis *in vivo *[34]. Thus, like PECAM-KO mice (which are fertile and viable; Duncan *et al*., 1999), CIB1-KO mice may also have an underlying endothelial permeability defect that does not manifest overtly, but affects pathological tumor growth. Such defects in CIB1-KO ECs would play a large part in the increased gross and microscopic allograft tumor bleeding observed in our study. Further studies are currently underway to characterize the nature of these defects and the implications they have on both physiological and other pathological processes.

## Conclusions

Taken together, our findings support a model in which CIB1 plays an important role in regulating tumor viability, growth, and angiogenesis. We propose that CIB1 mediates these effects at least in part through its previously characterized role in PAK1-ERK1/2-MMP-2 signaling. The fact that more necrosis and bleeding was noted in melanoma tumors in CIB1-KO mice, where there is a relative lack of MMP-2, suggests that this mechanism may be a major pathway mediating the observed effects in this study. Hence the data herein establish a regulatory role for CIB1 in tumor growth and pathological tumor-induced angiogenesis.

## List of Abbreviations

CIB1: calcium and integrin binding protein 1; PAK1: p21 activated kinase 1; MMPs: matrix metalloproteinases; KO: knock out; FAK: focal adhesion kinase; ECs: endothelial cells; VEGF: vascular endothelial growth factor; FGF-2: basic fibroblast growth factor 2; WT: wild type; UNC-CH: University of North Carolina Chapel Hill; H&E: Hematoxylin and Eosin; MT: Masson's trichrome; IgG: immunoglobulin G.

## Competing interests

The authors declare that they have no competing interests.

## Authors' contributions

**MAZ **planned and carried out most of the experiments and wrote the manuscript.

**WY **assisted with managing animal breeding and with critical input on experiments.

**DC **assisted with the Doppler perfusion experiments.

**JEF **contributed to the planning and design of experiments and with manuscript revision.

**LVP **contributed to data interpretation, manuscript preparation and revision. All others authors have read and approved the final manuscript.

## Authors' information

**MAZ **carried out these studies in partial fulfillment for the PhD degree while enrolled in the MD/PhD program at UNC-CH. MAZ is currently a vascular surgery resident at Stanford University Medical Center.

**WY **created the CIB1 knockout mice and is an expert molecular biologist.

**DC **is a postdoctoral research associate in the Department of Cell and Molecular Physiology at UNC-CH.

**JEF **is a full professor in the Department of Cell and Molecular Physiology at UNC-CH. He is expert in physiologic and genetic regulation of collaterogeneis and angiogenesis during development and disease, as well as in vascular wall growth and remodeling.

**LVP **was the thesis advisor for MAZ. She is also full professor and Chair of the Department of Biochemistry and Biophysics at UNC-CH. Her lab originally discovered CIB1 and she is an expert on integrin mediated adhesion and signal transduction in vascular cells.

## Supplementary Material

Additional file 1 **Carcinoma tumors in CIB1-KO mice have less areas of high perfusion**. Carcinoma tumors in CIB1-KO mice have reduced perfusion. Superficial hind-limb ventral adductor thigh regions, where tumor cells were injected, were monitored with noninvasive scanning laser-Doppler perfusion imaging (model LD12-IR, Moor Instruments; modified for high resolution and depth of penetration (2 mm) with an 830 nm-wavelenght infrared 2.5 mW laser diode, 100 μm beam diameter, and 15 kHz bandwidth). Isofluorane anesthesia and rectal temperature (37 ± 0.5°C) were maintained the same for all measurement days and among animals. (a) Laser-Doppler perfusion imaging at the site of tumor injection pre- (day 1), and day-7 and -14 after tumor cell injection. Image scans are assembled from the mean Doppler velocity (in perfusion units, PU) for each 100 × 2000 μm voxel in the scan area. (b) Regions of interest (ROI) were drawn around tumor perimeters, to derive a tumor area, and determine what percentage of the area had a detectable Doppler signal. For every given ROI, a tumor perfusion index was calculated using: (Average PU) × (ROI area) × (% ROI with detectable Doppler signal)/10,000. Error bars are ± SEM (*n *= 8-9 mice per group). Click here for file
