# Supplementary figures and images for: Tumor growth and angiogenesis is impaired in CIB1 knockout mice
Source: J Angiogenes Res. 2010 Aug 30;2:17. doi: 10.1186/2040-2384-2-17 (PMC2941741; doi:10.1186/2040-2384-2-17)

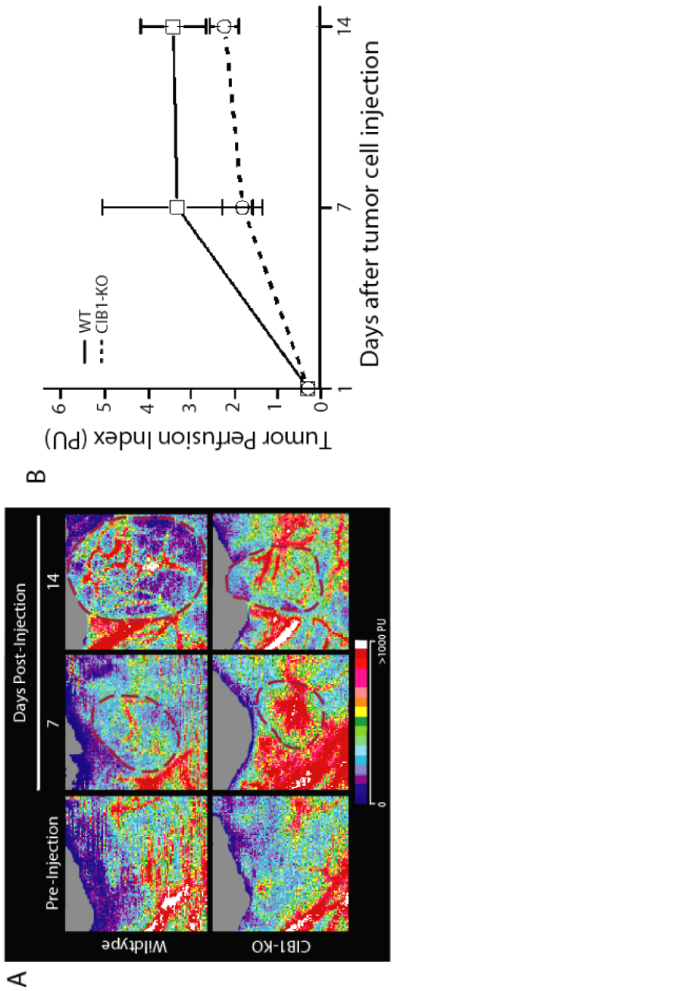

Supplement: Additional file 1 — Carcinoma tumors in CIB1-KO mice have less areas of high perfusion. Carcinoma tumors in CIB1-KO mice have reduced perfusion. Superficial hind-limb ventral adductor thigh regions, where tumor cells were injected, were monitored with noninvasive scanning laser-Doppler perfusion imaging (model LD12-IR, Moor Instruments; modified for high resolution and depth of penetration (2 mm) with an 830 nm-wavelenght infrared 2.5 mW laser diode, 100 μm beam diameter, and 15 kHz bandwidth). Isofluorane anesthesia and rectal temperature (37 ± 0.5°C) were maintained the same for all measurement days and among animals. (a) Laser-Doppler perfusion imaging at the site of tumor injection pre- (day 1), and day-7 and -14 after tumor cell injection. Image scans are assembled from the mean Doppler velocity (in perfusion units, PU) for each 100 × 2000 μm voxel in the scan area. (b) Regions of interest (ROI) were drawn around tumor perimeters, to derive a tumor area, and determine what percentage of the area had a detectable Doppler signal. For every given ROI, a tumor perfusion index was calculated using: (Average PU) × (ROI area) × (% ROI with detectable Doppler signal)/10,000. Error bars are ± SEM (n = 8-9 mice per group). [file 2040-2384-2-17-S1.PNG]
